# NET Models Meeting 2024 white paper: the current state of neuroendocrine tumour research models and our future aspirations

**DOI:** 10.1530/EO-24-0055

**Published:** 2024-12-19

**Authors:** Po Hien Ear, Ilaria Marinoni, Talya Dayton, Rachael Guenter, Dawn E Quelle, Anna Battistella, Floryne O Buishand, Suganthi Chittaranjan, Yi-Cheih Nancy Du, Ines Marques, Natalia S Pellegata, Samira M Sadowski, Amit Tirosh, Simon April-Monn, Cinzia Aurilia, Renata Jaskula-Sztul, Maria Jesús Baena Moreno, Simone Donati, Katherine A English, Maria Almudena Hernandez Llorens, Harry Hodgetts, Francesca Marini, Maria Martins, Gaia Palmini, Beatriz Soldevilla, Jörg Schrader, Rajesh V Thakker, Kate E Lines

**Affiliations:** ^1^ Department of Surgery, University of Iowa, Iowa City, Iowa, USA; ^2^ Institute of Tissue Medicine and Pathology, University of Bern, Bern, Switzerland; ^3^ European Molecular Biology Laboratory (EMBL) Barcelona, Tissue Biology and Disease Modelling, Barcelona, Spain; ^4^ Department of Surgery, School of Medicine, University of Alabama at Birmingham, Birmingham, Alabama, USA; ^5^ Department of Neuroscience and Pharmacology, University of Iowa, Iowa City, Iowa, USA; ^6^ Pancreatic Surgery Unit, Pancreas Translational and Clinical Research Center, IRCCS San Raffaele Scientific Institute, Vita-Salute San Raffaele University, Milan, Italy; ^7^ Malignant B Cells Biology and 3D Modelling Unit, Experimental Oncology Department, Vita-Salute San Raffaele University, Milan, Italy; ^8^ Department of Clinical Science and Services, Royal Veterinary College, Hatfield, UK; ^9^ Department of Genome Sciences Centre, BC Cancer, Vancouver, British Columbia, Canada; ^10^ Department of Pathology and Laboratory Medicine, Weill Cornell Medicine, New York, New York, USA; ^11^ Institute of Anatomy, University of Bern, Bern, Switzerland; ^12^ Department of Biology and Biotechnology, University of Pavia, Pavia, Italy; ^13^ Institute for Diabetes and Cancer, Helmholtz Munich, Munich, Germany; ^14^ Endocrine Surgery Section, Surgical Oncology Program, Center for Cancer Research, NCI, NIH, Bethesda, Maryland, USA; ^15^ ENTIRE – Endocrine Neoplasia Translational Research Center, Sheba Medical Center, and Tel Aviv University Faculty of Medicine, Tel Aviv, Israel; ^16^ Department of Pathology and Molecular Pathology, University Hospital Zurich, Zurich, Switzerland; ^17^ Associazione Italiana Neoplasie Endocrine Multiple di tipo 1 e di tipo 2 (AIMEN 1 e 2), Sondrio, Italy; ^18^ Center of Experimental Oncology, Gastrointestinal and Neuroendocrine Tumors Research Group, Research Institute Hospital 12 de Octubre (i+12), Madrid, Spain; ^19^ OCDEM, Radcliffe Department of Medicine, University of Oxford, Churchill Hospital, Oxford, UK; ^20^ Regenerative Medicine and Fibrosis Group, Institute for Liver and Digestive Health, University College London, Royal Free Campus, London, UK; ^21^ FIRMO Foundation (Italian Foundation for the Research on Bone Diseases), Florence, Italy; ^22^ Department of Genetics, Physiology and Microbiology, Faculty of Biology, Universidad Complutense de Madrid (UCM) Madrid, Spain; ^23^ Department of Medicine, University Medical Center Hamburg-Eppendorf, Hamburg, Germany; ^24^ Oxford NIHR Biomedical Research Centre, Oxford University Hospitals Trust, Oxford, UK; ^25^ Centre for Endocrinology, William Harvey Research Institute, Barts and the London School of Medicine, Queen Mary University of London, London, UK; ^26^ School of Biological and Medical Sciences, Faculty of Health and Life Sciences, Oxford Brookes University, Oxford, UK

**Keywords:** *in vitro*, *ex vivo*, *in vivo*, consortium

## Abstract

Current models for the study of neuroendocrine tumours (NETs) are severely limited. While *in vitro* (e.g. cell lines), *ex vivo* (e.g. organoids) and *in vivo* (e.g. mice) models all exist, each has limitations. To address these limitations and collectively identify strategies to move the NET models field forward, we held an inaugural NET models meeting, hosted by our founding group: Dr Lines (Oxford), Prof. Quelle (Iowa), Dr Dayton (Barcelona), Dr Ear (Iowa), Dr Marinoni (Bern) and Dr Guenter (Alabama). This two-day meeting in Oxford (UK) was organised and supported by Bioscientifica Ltd and was solely dedicated to the discussion of NET models. The meeting was attended by ∼30 international researchers (from the UK, EU, Israel, USA and Canada). Plenary talks were given by Prof. Thakker, who summarised NET research over the past few decades, and Dr Schrader, who described the process and pitfalls of generating new cell lines. Eight researchers also presented their work on topics ranging from human cell 3D bioprinting to zebrafish models and included novel ideas and improvements on current concepts. This was followed by an interactive workshop, where discussion topics included a summary of currently available NET models, limitations of these models, barriers to developing new models, and how we can address these issues going forward. This white paper summarises the key points raised in these discussions and the future aspirations of the NET Models Consortium. The next meeting will take place in Oxford (UK) in 2025; contact contact@netcancerfoundation.com for more information.

## Introduction

Neuroendocrine neoplasms (NENs) are tumours that arise in the neuroendocrine cells of the body and commonly affect the pancreas, lung or small intestine (Chauhan *et al.* 2020). They can occur sporadically or as part of inherited tumour syndromes, such as multiple endocrine neoplasia type 1 (MEN1) (Frost *et al.* 2018). Although considered a rare tumour type, the incidence of NENs is rising globally, with approximately 8.8 per 100,000 people diagnosed in England and approximately 10 per 100,000 people diagnosed in the USA (Chauhan *et al.* 2020, Das & Dasari 2021, White *et al.* 2022). Notably, low-grade NENs are particularly challenging to study and treat clinically because of their unusually slow growth, which not only makes them unresponsive to many traditional therapeutics but also has impeded the development of much-needed patient-derived cell systems and animal-based NEN models for research.

NENs can be classified as well-differentiated neuroendocrine tumours (NETs) or poorly differentiated neuroendocrine carcinomas (NECs). The latter are highly aggressive tumours that are both biologically and clinically representative of more common cancer types, such as breast or lung adenocarcinomas, as they harbour mutations in classic tumour suppressor genes, including *TP53* (Rindi *et al.* 2018). NETs, on the other hand, are a more diverse and biologically distinct tumour type, which can be further subdivided into three different grades (G): G1, G2 and G3 (Rindi *et al.* 2018). G1 tumours are the least aggressive, whereas G3 the most aggressive, with Ki-67 index and mitotic count increasing across the grades (Rindi *et al.* 2018). Most NETs are low G1 and G2 lesions but can still metastasise. There are >20 genes described to be mutated in NETs, with the most common being menin 1 (*MEN1*), death-domain-associated protein (*DAXX*) and ATRX chromatin remodeler (*ATRX*), which occur in 40–70% of sporadic G1–G3 pancreatic NETs (PanNETs), and cyclin-dependent kinase inhibitor 1B (*CDKN1B*), which occurs in approximately 10% of sporadic G1–G3 small intestinal NETs (siNETs) (Jiao *et al.* 2011, Francis *et al.* 2013, Di Domenico *et al.* 2017, Scarpa *et al.* 2017, van Riet *et al.* 2021). Mutations in other genes, such as phosphatase and tensin homologue (*PTEN*), and those in the mammalian target of rapamycin (mTOR) signalling pathway have also been identified, predominately in PanNETs, but at lower frequencies (Jiao *et al.* 2011, Di Domenico *et al.* 2017, Maharjan *et al.* 2021, van Riet *et al.* 2021).

The only curative treatment for NENs of all grades is surgery; however, 20–50% of patients present with unresectable tumours at diagnosis such that surgery is not an option (Buicko *et al.* 2019, O'Dorisio *et al.* 2020, Roeyen *et al.* 2009). The first-line medical treatment for NENs is somatostatin analogues (SSAs, either octreotide or lanreotide), which functionally engage somatostatin receptors that are often highly expressed in NENs (Rinke *et al.* 2009, Caplin *et al.* 2014). While SSAs stabilise disease and reduce morbidities associated with excessive hormone secretion, they do not reduce disease burden (O'Dorisio *et al.* 2020, Zandee & de Herder 2018). Other medical therapies available to patients with NENs include the mTOR inhibitor everolimus, the tyrosine kinase inhibitor sunitinib and conventional chemotherapies. Although some of them show promise, e.g. temozolomide shows progression-free survival of ∼50% in pancreatic NETs (PanNETs), many show limited and mixed efficacy (O'Dorisio *et al.* 2020, Raymond *et al.* 2011, Yao *et al.* 2011, 2016, Zandee & de Herder 2018). Radiological treatments, including peptide receptor radionuclide therapy (PRRT), transarterial chemoembolisation, transarterial radioembolisation and radiofrequency ablation, are also an option for NEN patients. However, similar to medical therapies, although some show promise, e.g. PRRT shows progression-free survival of ∼40% in PanNETs, many have limited efficacy (O'Dorisio *et al.* 2020, Singh *et al.* 2024, Strosberg *et al.* 2017, Zandee & de Herder 2018), although there are promising anti-tumour effects of recently developed α-emitting NEN-targeted agents (Gape *et al.* 2024, Lee *et al.* 2024). Nonetheless, although the 5-year survival rate for patients with localised NETs is 78–93%, the 5-year survival of patients with metastatic disease is only 19–38% (Riihimäki *et al.* 2016). Improved and more effective targeted therapies that are developed specifically for NENs, and particularly NETs, are therefore required. In order to develop these therapies, improved preclinical models are also required. To address this, we held the inaugural NET models meeting in 2024, bringing together experts in the field to present their current unpublished work and discuss the current state of NET models, the limitations of these, the barriers to improving the models and aspirations for how models can be improved in the future. This white paper therefore summarises the presentations and discussions from this meeting.

## NET models meeting overview

More models of NETs are desperately needed to address the important basic and translational research questions about this disease, as those answers will ultimately help advance NET patient treatments. NETs provide a unique challenge as they are relatively rare, genetically and phenotypically diverse and nearly impossible to propagate under standard *in vitro* and *in vivo* conditions that work for common tumour types. As a consequence, generating NET models for scientific research that accurately recapitulate the patient tumours remains highly complex. This is true for *in vitro* models (predominantly, cell lines), *ex vivo* models (e.g. organoids) and *in vivo* models (e.g. mice). Indeed, currently available NET research models are few in number, with many being imperfect for the molecular biology or therapeutic studies being undertaken. To address these challenges, the NET Models Consortium was established in 2023. The aim of the consortium is to bring together researchers in the NET field to share expertise and experience, to discuss not only successes but also failed attempts, as well as to collaboratively develop novel models for the scientific community to use. In February 2024, the NET Models Consortium held their inaugural meeting in Oxfordshire, UK, called NET Models Meeting 2024. There were 28 delegates attending in total, including two plenary speakers, and eight oral research presentations.

Day 1 opened with a plenary talk by Prof. Rajesh Thakker from the University of Oxford (UK). He summarised some of the most recent NET research findings and how different models, predominantly the cell lines BON-1 and QGP1, as well as mouse NET models, have been utilised. Although this highlighted several excellent studies on genetics, epigenetics and novel treatment approaches for NETs, it also cemented the need for improved models to enable more clinically translatable research. Eight research presentations by a highly international group of speakers followed, each relating work currently being undertaken using different NEN (i.e. NET and NEC) models. Dr Amit Tirosh (Tel Aviv, Israel) described generation of a VHL-deficient pseudohypoxic pancreatic NEN (PanNEN) cell line, Dr Floryne Buishand (London, UK) outlined their use of canine insulinoma as a model for human malignant insulinoma, and Dr Suganthi Chittaranjan (Vancouver, Canada) discussed their work on exploring subgroup-defining biomarkers and therapeutic vulnerabilities in PanNEN models. Dr Anna Battistella (Milan, Italy) described their work on the development of new 3D *in vitro* models of pancreatic NETs (PanNETs), and Dr Samira Sadowski (Bethesda, USA) outlined their work on therapeutic screening in patient-derived organoids for gastroenteropancreatic NETs (GEP NETs). Three talks were focused on *in vivo* models: Dr Ines Marques (Bern, Switzerland) presented their work on zebrafish patient-derived xenograft (PDX) models as a tool for precision medicine and establishment of an *in vivo* pipeline to evaluate NETs, Prof. Natalia Pellegata (Munich, Germany) discussed *in vivo* models of different subtypes of paragangliomas, and Dr Yi-Cheih Nancy Du (New York, USA) spoke about a mouse model that allows the identification of metastatic factors by somatic gene transfer.

Day 2 of the meeting began with Dr Jörg Schrader (Husum, Germany) delivering his plenary talk on establishing innovative NET cell lines, in which he described the strategies his lab has used, as well as the pitfalls. Dr Schrader has successfully established at least two validated NET cell lines and concluded that defining the best culture conditions is of utmost importance to stimulate growth of NET cells, particularly as they, in most cases, lack classic oncogene activation. This was followed by a workshop to discuss the next steps and aspirations for improving NET models. This workshop was subdivided into three groups, *in vitro* models (chaired by Dr Kate Lines), *ex vivo* models (chaired by Dr Po Hien Ear) and *in vivo* models (chaired by Dr Ilaria Marinoni). The outcome of these discussions is outlined in the sections below.

## *In vitro* NET models

The term *in vitro* models refers to laboratory-cultured cells, either primary cells that have a defined lifespan or immortalised cell lines that can proliferate indefinitely. Cell lines are often genetically manipulated to proliferate or, in the case of cancer, may naturally harbour mutations that drive proliferation. For example, the human embryonic kidney cell line (HEK293) was generated by exposing HEK cells to adenovirus type 5, whereas HeLa cells generated from cervical cancer spontaneously proliferate in culture (Puck & Marcus 1955, Graham *et al.* 1977). The main advantage of cell lines is that they provide a low-cost, simple, high-throughput model that can be used to rapidly assess tumour cell biology and test novel therapies. Cell lines also provide a model in which the role and significance of certain molecular alterations seen in patients can be efficiently studied. For example, the expression of cancer-associated genes and proteins can be easily manipulated in cultured cells and allow functional outputs, such as tumour cell proliferation and survival, to be assessed in real time.

A number of NEN cell lines have been developed, and these are summarised in Table 1. Most cell lines commonly used were derived from human or rodent tumours. For PanNENs, the most widely used cell lines are BON-1 and QGP1, which were generated over thirty years ago. BON-1 cells were originally isolated from a metastatic tumour in the pancreas, while QGP1 cells were derived from a human pancreatic somatostatinoma (Kaku *et al.* 1980, Evers *et al.* 1991). Both grow as adherent, easily maintained cultures and harbour mutations that are commonly seen in NETS: BON-1 cells have a homozygous loss of cyclin-dependent kinase 2A (*CDKN1A*) and *CDKN2B*, and QGP1 cells have a mutation in *ATRX* (Hofving *et al.* 2018). However, whole exome sequencing has indicated that they may also harbour additional mutations that are typical of high-grade PanNETs or NECs, for example in *TP53* (Vandamme *et al.* 2015). BON-1 and QGP1 cells have been used for hundreds of studies ranging from basic molecular biology of NENs to drug screening, although neither is optimal for studying hormone secretion. Therefore, for investigating the effects on insulin secretion, rodent cell lines have been utilised, predominately the pancreatic beta cell-derived cell lines, MIN6 and INS1 (Miyazaki *et al.* 1990, Skelin *et al.* 2010). More recently, cell lines that are more representative of well-differentiated NETs have been described, including the human SPNE1, NT-18P and NT-3 cell lines (Benten *et al.* 2018, Lou *et al.* 2022, Viol *et al.* 2022). NT-3 cells have so far been the most widely used of these newer cell lines. NT-3 cells have been used, for example, to evaluate existing therapies and mutation-based targeted therapies (Viol *et al.* 2022, April-Monn *et al.* 2024), as well as to interrogate the role of cancer-associated fibroblasts (Amin *et al.* 2023).

**Table 1:** Currently available cell lines for NENs

Tissue of origin	Disease	Species	Cell line name	References
Adrenal	Pheochromocytoma	Rat	PC-12	Greene & Tischler (1976)
	Pheochromocytoma	Rat	PC-12 Adh	Greene & Tischler (1976)
Lung	Typical lung NET	Human	H727	Carney *et al.* (1985)
	Typical lung NET	Human	H835	Carney *et al.* (1985)
	Atypical lung NET	Human	H720	Carney *et al.* (1985)
Pancreas	PanNET	Human	BON-1	Evers *et al.* (1991)
	PanNET	Human	QGP1	Kaku *et al.* (1980)
	PanNET	Human	SPNE1	Lou *et al.* (2022)
	PanNET	Human	NT-3	Benten *et al.* (2018)
	PanNET	Human	NT-18P	Viol *et al.* (2022)
	PanNET liver metastasis	Human	NT-18LM	Viol *et al.* (2022)
	PanNET local recurrence	Human	NT-36	Viol *et al.* (2022)
	PanNET	Human	HuNET	Tillotson *et al.* (2001)
	PanNET	Canine	canINS	Capodanno *et al.* (2018)
	Insulinoma	Mouse	MIN6	Miyazaki *et al.* (1990)
	Insulinoma	Mouse	N134	Du *et al.* (2007)
	Insulinoma	Rat	INS1	Skelin *et al.* (2010)
Pituitary		Mouse	AtT20	Buonassisi *et al.* (1962)
Small bowel	Ileal NET	Human	GOT1	Kölby *et al.* (2001)

Similarly to PanNETs, a few lung NETs exist, including the widely used human H727 and H720 cells that represent typical and the more aggressive atypical lung NETs, respectively (Carney *et al.* 1985). There is just one vetted human cell line for small bowel NETs, GOT1 (Kölby *et al.* 2001). Rodent cell lines are also available for pheochromocytoma and pituitary adenomas; however, although the KAT45 (pheochromocytoma) and HP75 and GX (pituitary) cell lines were developed and used historically, they have either been lost over time or have extremely limited distribution, meaning that there are no widely available human cell lines (Buonassisi *et al.* 1962, Greene & Tischler 1976, Zhu *et al.* 2020, Karna *et al.* 2024). The extensive use of all of these cell lines in both therapeutic and molecular biology studies reflects their value to the NEN research community.

### Limitations of existing *in vitro* NET models

Although NEN cell lines exist, they have many limitations. Here we will discuss the limitations that were highlighted as being the most problematic. First, the existing cell lines are not representative of the genetic background in patient tumours and particularly are unrepresentative of the genetic drivers of hereditary conditions, such as MEN1. For example, there is no *MEN1* or *ATRX* knockout (KO) cell line available for any NEN subtypes seen in patients. In addition, many of the cell lines have either acquired or were selected for mutations in tumour suppressor genes, such as *TP53*, or oncogenes such as *KRAS*. This means that the cell lines are fast-growing, which is more representative of NECs rather than NETs. This limitation is exacerbated by the fact that the cell lines have been cultured by different groups over the past few decades, yielding highly variant clonal populations in which novel mutations have been acquired, a phenomenon highlighted by the increasing journal requests for cell line authentication and publications describing the characteristics and undertaking genetic analysis of the cell lines, for example Hofving *et al.* (2018), Luley *et al.* (2020) and Monazzam *et al.* (2020). Such diversity in NEN cell lines studied in different labs can impact data reproducibility. One way to overcome these limitations is to develop novel cell lines. Dr Jörg Schrader (Husum, Germany) presented his work establishing the well-differentiated NT-3 insulinoma cell line, derived from a lymph node metastasis in a PanNET patient. His team also generated NT-18P, NT-18LM and NT-36 PanNET cells from the primary tumour, liver metastasis and a local recurrence (12 months after initial surgery and chemotherapy treatment), respectively, from a patient with a G3 PanNET. Dr Amit Tirosh (Tel Aviv, Israel) also summarised their work on generating a VHL-deficient pseudohypoxic pancreatic NEN cell line (Telerman *et al.* 2023). All these cell lines, while representing an advance over previous models, are nonetheless limited by the fact that they originated from high-grade lesions and represent functional tumours. Most patient PanNETs are low-grade (G1 or G2) and non-functional, but no cell lines representing those types of tumours have been successfully established. That remains a major gap in the NET field.

It was also highlighted that the lack of hormone secretion from some of the ‘functioning’ human cell lines, e.g. BON-1 (Luley *et al.* 2020), hinders the molecular biology studies that can be undertaken. This combined with the fact that there is still a lack of knowledge on the optimal culture factors and conditions required for the existing cell lines further limits the use of these cells. Murine PanNET cells are available for examining insulin secretion (for example, MIN-6, INS-1 or N134 cells); however, these do not recapitulate the genetics seen in humans. During this meeting, Dr Floryne Buishand (London, UK) discussed the canine canINS cell line that was established from a canine insulinoma. As dogs more closely represent the genetics of humans than rodents, the generation of canine NET models may provide a novel and valuable platform for NET research, especially since NETs arise spontaneously in dogs in contrast to genetically induced NETs in rodents (Capodanno *et al.* 2022).

### Current barriers for the use and generation of novel NET *in vitro* models

We also discussed the barriers to developing new NET cell lines. It was concluded that the biggest barrier is ready access to the quantities of patient tumour tissue needed to generate cell lines. Many researchers do not have direct access to surgical theatres or any links to clinical colleagues, and therefore, access to fresh samples is limited. Those researchers that do have access to samples reported a lack of interest in research from surgeons and pathologists. This can result in missed samples due to logistical planning, for example, not being told about a surgery or surgeries ending late in the day such that tumour specimens could not be released to non-clinical staff. Some researchers do have good surgical links and are able to routinely access NEN tissue; however, there is no defined protocol for tissue collection and storage. Thus, different laboratories will handle samples in different ways, resulting in protocols for cell line generation that are not readily transferable. Finally, it is both administratively and physically complex to share patient tissue across groups or institutes, and especially complicated and costly to share material internationally. This means that even protocols that use cryo-frozen tissue cannot be implemented by groups other than those who have directly collected the material. Even if samples are acquired and protocols for cell line establishment undertaken, cells from low-grade NETs have an extremely slow growth rate. This makes both establishing and maintaining the cells extremely challenging. A major problem in this regard is the ‘contamination’ of samples with stromal cells. As fibroblasts have an at least 10× to 100× faster growth rate than NET cells, this warrants elaborated selection strategies to achieve pure tumour cell cultures, for example sequential trypsinization and cultivation under low-adherent conditions (Benten *et al.* 2018). A final major barrier is the lack of obligation to deposit cell lines into a publicly available repository (for example ATCC), as well as the lack of funds for undertaking this deposit. This means that researchers commonly transfer cell lines using material transfer agreements (MTAs), which can be time-consuming and/or not accepted by certain institutions, thereby restricting distribution.

## *Ex vivo* NEN models

We define NEN *ex vivo* models as models with high levels of resemblance to surgically resected NEN samples in terms of tumour heterogeneity. This category comprises patient-derived tumour organoid (PDTO) cultures, tumoroid or spheroid cultures and complex multi-cell type systems. PDTO cultures can be grown and expanded in culture indefinitely and are therefore defined as long-term cultures. In contrast, most tumoroid or spheroid cultures and complex multi-cell type bioreactor systems have a limited *in vitro* lifetime in terms of growth and expansion and are thus defined as short-term cultures.

Long-term PDTO culture systems use a completely defined culture medium consisting of a cocktail of growth factors designed to recapitulate the cellular signalling environment of the native tumour microenvironment (Clevers 2016). This growth factor-rich medium enables the establishment of PDTOs from early-stage and low-grade tumours (Fujii *et al.* 2016, Boretto *et al.* 2019, Kopper *et al.* 2019). Thus, successful derivation of PDTOs from NETs could be facilitated by prior knowledge of the growth requirements of either NETs themselves or their presumed cells of origin, neuroendocrine cells. Long-term PDTO cultures have been established for low-grade lung NETs (also known as pulmonary carcinoids), with a reported success rate of 37% (Dayton *et al.* 2023). Underscoring the importance of growth factor signalling for NETs, lung NET PDTOs are largely dependent on EGF in the medium for their growth. Long-term PDTO cultures have also been established for G3 NETs: one from a biliary tract NET, one from a PanNET, and one from a duodenal NET (Kawasaki *et al.* 2020). The duodenal NET PDTO is reported to be EGF dependent, and the pancreatic NET carries an amplification of *ERBB2*, suggesting that EGF signalling may also be important for some PanNETs. A PDTO model of a G2 small intestinal NET has also been reported (D'Agosto *et al.* 2023). NET PDTOs display an extremely slow growth rate requiring culture expansions in the range from every 10 days to every 3 months (D'Agosto *et al.* 2023, Dayton *et al.* 2023).

Certain categories of NETs, such as the gastroenteropancreatic (GEP) NETs, which include PanNETs and NETs of the gastrointestinal tract, remain challenging to culture long term. Only 4–12% of GEP NET PDTO models have been successfully cultured beyond passage 5 or over a 6-month period, which pales in comparison with other NET and NEC PDTO models (D'Agosto *et al.* 2023, Dayton *et al.* 2023, Kawasaki *et al.* 2020). As an alternative, short-term NET spheroid models have been established and demonstrated to express NET markers (Ear *et al.* 2019, April-Monn *et al.* 2021, Gillette *et al.* 2021). The main advantage of short-term NET spheroid culture is the high success rate of establishment, which ranges from 85 to 90% (April-Monn *et al.* 2021, Gillette *et al.* 2021). The methodology of culture is similar to long-term NET PDTO cultures, where the emphasis is on the isolation of NET cells from fresh or cryo-preserved NET samples for embedding in an extracellular matrix. A major difference is in the medium composition (Table 2). Short-term cultures can be grown in either stem-cell-based medium or fetal bovine serum (FBS)-containing medium supplemented with additional growth factors and vitamins (EGF, FGF, PIGF, IGF-1, insulin or nicotinamide). FBS-containing medium formulations are less expensive compared to stem cell media and allow the usage of short-term NET *ex vivo* models for drug testing by many research laboratories (Ear *et al.* 2019, April-Monn *et al.* 2021, Gillette *et al.* 2021, April-Monn *et al.* 2024). Since well-differentiated NET cells are slow-growing and require 7–14 days to divide, the short-term *ex vivo* model offers an advantage to bypass the long wait time for growth as it can immediately be used in drug testing experiments (Ear *et al.* 2019, April-Monn *et al.* 2021). Current published methods use 3,000–5,000 isolated NET cells per well, and up to six drugs or drug combinations can be tested at various concentrations (Ear *et al.* 2019, April-Monn *et al.* 2021, Gillette *et al.* 2021, April-Monn *et al.* 2024). Larger collections of GEP NET short-term spheroid drug screening studies with 17- and 14-NET patient spheroids screened with different libraries of compounds are underway. With improved sensitivity of detection assays and instrumentation, several research laboratories are currently developing novel methods to use as little as 500 cells per well of GEP NETs for high-throughput drug screening for precision medicine.

**Table 2:** Summary of culture media for NEN spheroids, organoids and tissues.

Culture type	Manuscripts	Media compositions
Spheroids	Ear *et al.* (2019)	DMEM/F12, 10% FBS, 1% penicillin/streptomycin, 1% glutamine, 10 mM nicotinamide, 10 μg/mL insulin
	April-Monn *et al.* (2021)	DMEM/F12, 5% FBS, HEPES 10 mM, 1% l-glutamine (200 mM), 1% penicillin (100 U/mL), 1% streptomycin (0.1 mg/mL), 1% amphotericin B (0.25 mg/mL), 20 ng/mL EGF, 10 ng/mL bFGF, 100 ng/mL PlGF, 769 ng/mL IGF-1
	Gillette *et al.* (2021)	DMEM/F12, 10% FBS, 1% penicillin/streptomycin, +1% GlutaMAX, 10 mM HEPES + EGF
Organoids	Kawasaki *et al.* (2020)	Advanced DMEM/F12 supplemented with penicillin/streptomycin, 10 mM HEPES, 2 mM GlutaMAX, 1× B27, 10 nM gastrin I and 1 mM *N*-acetyl cysteine. A complete medium was prepared by supplementing the basal culture medium with the following niche factors: 50 ng/mL mouse recombinant EGF, 50 ng/mL human recombinant FGF-2, 100 ng/mL human recombinant IGF-1 (BioLegend), 100 ng/mL mouse recombinant noggin (PeproTech), 1 mg/mL recombinant human R-spondin-1 (R&D), 25% afamin–Wnt-3A serum-free conditioned medium and 500 nM A83-01
	D'Agosto *et al.* (2023)	Advanced DMEM/F12 medium supplemented with 10 mM HEPES, GlutaMAX, Primocin (1 mg/mL, InvivoGen), *N*-acetyl-l-cysteine (1.25 mM), Wnt3a-conditioned medium (50% v/v), R-spondin-1-conditioned medium (10% v/v), recombinant Noggin (100 ng/mL), epidermal growth factor (EGF, 50 ng/mL), gastrin (10 nM), fibroblast growth factor 10 (FGF10, 100 ng/mL), nicotinamide (10 mM) and A83-01 (0.5 μM))
	Dayton *et al.* (2023)	Advanced DMEM/F12 supplemented with 1× GlutaMAX, 10 mM HEPES, penicillin–streptomycin, Primocin, 1% Noggin-conditioned medium, 20% of RSPO1-conditioned medium (made in-house), 1× B27 supplement, 1.25 mM *N*-acetyl cysteine, 3 μM CHIR, 1 μM prostaglandin E2, 0.005 μg/mL FGF2, 10 μM ROCK inhibitor, 500 nM A83-01 and 3 μM p38 inhibitor SB202190. All lung NET organoids and some LCNEC organoids were grown in media additionally supplemented with 0.05 μg/mL EGF
Tissue	Herring *et al.* (2021)	Phenol red-free DMEM/F12 supplemented with 10% FBS and penicillin/streptomycin

FBS, fetal bovine serum.

In addition to NEN organoid and spheroid models, which focus on 3D culture of the tumour cells, innovative 3D models with increased complexity are actively being developed. A study from colleagues at the University of Alabama successfully demonstrated the feasibility of maintaining patient PanNETs in culture in bioreactor chambers for 21 days and propagating them to secondary chambers for an additional 9 days while maintaining NET markers (Herring *et al.* 2021). Other efforts for co-culturing NET PDTOs and cancer-associated fibroblasts or endothelial cells, including the usage of hydrogel-based extracellular matrices, are underway. The development of tissue engineering technology based on 3D printing could also help improve current models and scale up production for usage in drug testing experiments.

### Limitations of existing *ex vivo* NET models

While long-term NET PDTOs can be expanded in culture indefinitely, a major limitation is their slow growth rate because they require a significant investment in terms of time and resources for expansion and long-term maintenance. This makes these NET PDTO models most suitable for mechanistic studies, targeted drug screens or CRISPR/Cas9 experiments that can be conducted within reasonable timeframes. Nonetheless, the number of available NET PDTOs is limited. We are still in search of the optimal growth factor(s) and small molecule(s) cocktail to reliably extend the life of cultured NET cells. In contrast, short-term GEP NET spheroid models, which last 1–3 months, fit well with medium-scale drug screening studies. These short-term models have a significant drawback, however, in that they are not suitable for genetic studies. This is because genetic editing methods, such as CRISPR/Cas9, require long-term cultures to allow for the selection and growth of genetically altered cells.

### Current barriers for the use and generation of novel NET *ex vivo* models

Many of the barriers faced by *ex vivo* models are similar to those mentioned for *in vitro* NET models with regard to access to tumour samples and IRB protocols that limit sharing of patient tissues. Especially in cases where different informed consent forms are used by different studies or different hospitals providing tissue for the same study, teasing apart what is allowed with samples from one hospital versus another with regard to follow-up studies and sharing may be a time-consuming task that can involve multiple ethical committees and assessments.

## *In vivo* NEN models

*In vivo* models, or animal models, are crucial to study the impact of cancer on the entire body of a complex, living organism. Animal models enable researchers to study the mechanisms of cancer development, progression and spread to distant organs and provide a platform for the discovery and evaluation of new therapies. *In vivo* models are particularly important for endocrine cancers as they allow the assessment of hormone secretion effects, as well as the study of rare endocrine tumour syndromes, such as MEN1, in which multiple tumours occur in different organs simultaneously.

In the field of NENs, several animal models have been developed across multiple species, including mice, rats, dogs, zebrafish and fruit flies (for example, see Vitale *et al.* (2014), Lines *et al.* (2016), Sedlack *et al.* (2022), Forsythe *et al.* (2023) and Karna *et al.* (2024)). As those publications have already comprehensively reviewed existing NEN models, we will not revisit them all here, although Table 3 provides a brief overview. These models have been instrumental in understanding NEN biology, mechanisms of NEN progression, and systemic effects of hormone hypersecretion and in identifying novel treatments and targets. The most widely used NEN models are genetically engineered mouse models (GEMMs), which are ideal for studying the biology of early disease progression. Many KO GEMMs of *Men1* exist, including constitutive and conditional models, and they represent a valuable model to study NEN syndromes, for example MEN1 (Crabtree *et al.* 2001, 2003, Bertolino *et al.* 2003, Loffler *et al.* 2007, Harding *et al.* 2009). Mutations in *MEN1*, which encodes the menin protein, trigger NEN formation both in mice and in humans, and therefore, the *Men1*-KO GEMMs recapitulate the NEN development observed in humans, affecting parathyroids, pancreas and pituitary (Crabtree *et al.* 2001, 2003, Bertolino *et al.* 2003, Loffler *et al.* 2007, Harding *et al.* 2009, Lines *et al.* 2017). These models have been used to understand the roles of both *MEN1* and menin and to identify avenues for targeting dysregulated MEN1 function.

**Table 3:** Summary of the types of existing *in vivo* NEN models.

Model type	Species					
	Drosophila	Zebrafish	Mouse	Rat	Dog	Frog
GEMM	Yes	Yes	Yes	Yes	No	Yes
Spontaneous	No	No	No	Yes	Yes	No
Xenograft	No	Yes	Success rate around 10%	Yes	No	No

Despite not always faithfully reflecting the human tumours, existing GEMMs have been extremely valuable for studying and understanding the biological landscape of NENs. While *Men1*-KO mice were specifically generated to target a driver mutation equivalent to a human counterpart, other animal models have been established, which develop NENs through different mechanisms. For example, the RIP1-Tag2 model is a transgenic model expressing the SV40 large T antigen under the control of the rat insulin promoter, where the function of the p53 and RB tumour suppressor proteins is inhibited by the T antigen in pancreatic beta cells (Hanahan 1985). RIP1-Tag2 mice develop aggressive, fast-growing and insulin-secreting pancreatic NECs (PanNECs) around 16 weeks of age (Hanahan 1985). Although the biology of these tumours does not recapitulate the slow-growing nature of G1 or G2 human PanNETs, this model has been widely used to study angiogenesis and tumour progression and to identify new potential treatments, some of which are now used clinically for patients with PanNETs (Hanahan *et al.* 1996, Bill *et al.* 2015).

An alternative to GEMMs are models in which a tumour develops due to spontaneously arising mutations in the animal. The MENX rat model, presented at the 2024 NET Models Meeting by Prof. Pellegata (Munich, Germany), carries a spontaneous mutation in the *Cdkn1b* gene (encoding the p27 tumour suppressor) and develops NENs in pituitary gland, adrenal glands, pancreas and thyroid gland with high penetrance within the first 8–9 months (Piotrowska *et al.* 2004). Tumours in this model share biochemical, physiological and molecular characteristics with the cognate human NETs. Germline mutations in the human homologue *CDKN1B* cause the MEN4 syndrome (Pellegata *et al.* 2006, Molatore *et al.* 2010, Lee *et al.* 2013). During the meeting, it was also highlighted that dogs spontaneously develop insulinomas and the incidence rate of insulinomas in dogs is ten times higher than in humans (Capodanno *et al.* 2022). The advantages of working with an insulinoma model in dogs include the fact that dogs live together with humans, are thus exposed to the same environmental factors and may share their diet with humans. In addition, spontaneous canine insulinomas offer intact host immunity, as well as natural tumour heterogeneity and microenvironment. When diagnosed with an insulinoma, dogs are treated as humans are, with surgery being the recommended first-line care. In addition, spontaneous canine insulinomas develop in different dog breeds, thus encompassing the heterogeneity observed in human patients, which certainly is a limitation of inbred rodent strains. However, further investigation into the genetic background of these tumours to assess whether they share the same drivers as human patients is warranted, to establish the full value of this model, especially in terms of phenocopying the human disease.

PDX transplantation of NENs into immunocompromised animals represents a third possible *in vivo* model. Unfortunately, NETs have a low capacity to engraft and require long time periods (up to 2 years) to grow. NEC PDX models have higher engraftment rates, and several models have been reported in recent years, for example Tran *et al.* (2022). These models are valuable for assessing novel treatments in the presence of a more representative microenvironment and have particularly been utilised for the study of PRRT. Although the use of zebrafish has previously been described in NENs (Vitale *et al.* 2014), a new interesting technique was also presented at the meeting consisting of the transplantation of SiNETs and PanNETs into zebrafish embryos. This model has already been widely used for other cancer types (Marques *et al.* 2009, Fior *et al.* 2017). To develop a NEN PDX, tumour cells are injected into the yolk sac of zebrafish embryos, where they are able to survive, recruit vessels, migrate to the tail vein and even form micro-metastases in the liver (unpublished data). Zebrafish embryos bearing NENs can be treated with anti-tumour drugs, and the effects of the drugs on the tumour cells’ behaviour can be effectively measured. In addition, zebrafish provide a model in which live imaging can be performed to follow the tumour cells.

### Limitations of existing *in vivo* NET models

The currently available *in vivo* models of NENs have several limitations. A major limitation is the lack of models bearing some of the relevant driver mutations seen in human patients. For example, there are no animal models representing the most common mutations occurring in PanNETs: *DAXX* and *ATRX*. GEMMs bearing either of these mutations do not develop PanNETs (Wasylishen *et al.* 2020, Sun *et al.* 2022). A recent mouse model combining *Atrx*, *Men1* and *Pten* deletion demonstrated development of tumours; however, they were aggressive PanNECs rather than NETs (Fuentes *et al.* 2024). Another major limitation is that the majority of available mouse models for PanNETs develop insulinomas. Only one model currently exists for non-functioning PanNETs, which represent the majority of PanNETs diagnosed in patients and have worse prognoses. Carter and coworkers generated an inducible, conditional mouse model of PanNETs by hyperactivation of CDK5 in β cells, which promoted the development of both functional and non-functional well-differentiated tumours (Carter *et al.* 2021).

To investigate the molecular networks that drive tumour progression and metastasis, Dr Yi-Cheih Nancy Du (New York, USA) presented their work on the development of a bitransgenic mouse model, *RIP-Tag*; *RIP-tva*, in which both the SV40 large T antigen and the receptor for subgroup A avian leucosis virus (*tva*) are expressed in pancreatic β cells under the control of the rat insulin promoter (Du *et al.* 2007, Zhang *et al.* 2017). As such, genetic alterations can be introduced *in vivo* into pancreatic β cells by infection with avian retroviral vectors harbouring desired genetic alteration. Using this model, Dr Du’s lab demonstrated that Bcl-xL, RHAMM^B^ and miR-431 promote PanNET metastasis (Du *et al.* 2007, 2011, Choi *et al.* 2016, 2019, Zhang *et al.* 2017, 2020). Dr Du also derived several insulinoma cell lines, including N134 (Du *et al.* 2007), from PanNETs in *RIP-Tag*; *RIP-tva* mice for an *in vitro* study. Avian retroviral vectors can infect these murine PanNETs cell lines with high efficiency to overexpress or knock down candidate genes (Zhang *et al.* 2017).

A major limitation to research involving GEMMs is the expense and time-consuming nature of the work, especially for slowly growing NETs that may take 1–2 years to form *in vivo*. In some cases, specialised equipment may be required to image and track tumour growth non-invasively within the pancreas, for instance, the accessibility and cost of which may be prohibitive to researchers. While a key advantage of GEMMs is the presence of an intact immune system, xenograft models suffer from the inherent lack of an immune system in the host mice. This means that interactions between tumour cells and the tumour microenvironment (including immune cells) cannot be adequately addressed using PDX mouse models, and they fail to accurately mimic human disease. Furthermore, the cells used in PDX models are generally highly mutated and therefore are more representative of NECs, rather than NETs, once again failing to recapitulate the biological status of patient NETs. Finally, experimental work performed using inbred mouse strains contributes to a lack of reproducibility across different strains and even laboratories.

### Current barriers for the use and generation of novel NET *in vivo* models

The 2024 NET Models Meeting also had discussions about the barriers to generating new *in vivo* NET models. This highlighted two main barriers, the first being that generating GEMMs or other animal models is costly and the second being that NETs have a limited capacity to proliferate *in vivo*, making the delay in NET development lengthy. Thus, the delay in tumour growth can become extremely long, wherein some PanNETs take up to two years to develop. This combined with the cost makes it very expensive to fully characterise new models and confirm whether they indeed develop the required NETs. Other animal models are also possible, for example zebrafish. These are, however, less commonly used, and therefore, there is a lack in the field of appropriate expertise and equipment.

## Future aspirations

The main aim of the NET Models Consortium is to improve the availability and support the generation of new, more appropriate NET models. Based on the discussions at this meeting, the following practical steps towards achieving this goal were proposed. First, any cell lines should be deposited into a repository (for example ATCC). This would not only improve access to the cell lines but also data reproducibility as it would limit the number of sub-clones generated. It would also provide a reference genome for any authentication protocols. The limiting factor in such an effort may be accessing funds as the deposition often comes at a cost. It was therefore concluded that this should be considered when submitting grant applications. Similarly, there should be more transparency in declaring any acquired mutations or characteristics (for example, that may be identified in sequencing studies or biomarker screens) in cell line sub-clones when publishing results. In addition, co-culture or 3D culture models should be considered alongside standard 2D cell line culture protocols as cells often behave differently in these conditions. Indeed, co-culture systems may more closely recapitulate the *in vivo* tumour environment, which may improve the translation of *in vitro* work into *in vivo* studies.

For all studies, it was also concluded that collaboration is key. This includes making data and protocols available, as well as sharing knowledge, reagents and materials (including cells and tissues). The aim of this meeting is to foster productive collaborations by bringing together researchers with the necessary skills and knowledge. In addition, collaborations with researchers from other disciplines should be encouraged as that may help solve inherent challenges faced when working with NENs. The aims of these collaborations are to standardise protocols for collection of primary material; validate findings across laboratories; generate new animal models of NETs that highly resemble disease in humans, including considering expansion to other species besides the classically used rodents; share animal-related resources to reduce duplication (in line with the ‘3R’ principle: replace, reduce and refine); and apply for joint funding to further support the exchange of models/materials to impact translational research of NETs and thereby ultimately improve NET patients’ management and survival.

## Conclusions

In conclusion, high-quality *in vitro*, *ex vivo* and *in vivo* NET models that accurately mimic the patient tumours are still limited and in great demand for NET research. The NET Models Consortium has brought together international experts in this field to discuss the limitations of current models and provide tangible and deliverable aims to improve them in a collaborative manner. A framework has also been developed to continue this work at future meetings, with the next to be held in Oxford in 2025. By providing investigators with an annual opportunity to present and discuss their unpublished data, including negative results, with experts in the field, it is envisaged that the NET Models Consortium meetings will foster productive collaborations required for more efficient data and resource sharing. We expect that such interactions will greatly facilitate future advances in NET model development and research.

## Declaration of interest

K E Lines is an Associate Editor of *Endocrine Oncology*. K E Lines was not involved in the review or editorial process for this paper, on which she is listed as an author.

## Funding

This work did not receive any specific grant from any funding agency in the public, commercial or not-for-profit sector.

## Author contribution statement

PHE, IM, TD, RG, DEQ are NET Models Consortium subgroup leads and contributed equally to this manuscript. KEL is the NET Models Consortium lead.

## References

[bib1] Amin T, Viol F, Krause J, et al. 2023 Cancer-associated fibroblasts induce proliferation and therapeutic resistance to everolimus in neuroendocrine tumors through STAT3 activation. Neuroendocrinology 113 501–518. (10.1159/000528539) 36473454

[bib2] April-Monn SL, Wiedmer T, Skowronska M, et al. 2021 Three-dimensional primary cell culture: a novel preclinical model for pancreatic neuroendocrine tumors. Neuroendocrinology 111 273–287. (10.1159/000507669) 32241015

[bib3] April-Monn SL, Kirchner P, Detjen K, et al. 2024 Patient derived tumoroids of high grade neuroendocrine neoplasms for more personalized therapies. NPJ Precis Oncol 8 59. (10.1038/s41698-024-00549-2) 38429350 PMC10907580

[bib4] Benten D, Behrang Y, Unrau L, et al. 2018 Establishment of the first well-differentiated human pancreatic neuroendocrine tumor model. Mol Cancer Res 16 496–507. (10.1158/1541-7786.mcr-17-0163) 29330294

[bib5] Bertolino P, Tong WM, Galendo D, et al. 2003 Heterozygous Men1 mutant mice develop a range of endocrine tumors mimicking multiple endocrine neoplasia type 1. Mol Endocrinol 17 1880–1892. (10.1210/me.2003-0154) 12819299

[bib6] Bill R, Fagiani E, Zumsteg A, et al. 2015 Nintedanib is a highly effective therapeutic for neuroendocrine carcinoma of the pancreas (PNET) in the Rip1Tag2 transgenic mouse model. Clin Cancer Res 21 4856–4867. (10.1158/1078-0432.ccr-14-3036) 26206868

[bib7] Boretto M, Maenhoudt N, Luo X, et al. 2019 Patient-derived organoids from endometrial disease capture clinical heterogeneity and are amenable to drug screening. Nat Cell Biol 21 1041–1051. (10.1038/s41556-019-0360-z) 31371824

[bib8] Buicko JL, Finnerty BM, Zhang T, et al. 2019 Insights into the biology and treatment strategies of pancreatic neuroendocrine tumors. Ann Pancreat Cancer 2 12. (10.21037/apc.2019.06.02) 31535089 PMC6750261

[bib9] Buonassisi V, Sato G & Cohen AI 1962 Hormone-producing cultures of adrenal and pituitary tumor origin. Proc Natl Acad Sci U S A 48 1184–1190. (10.1073/pnas.48.7.1184) 13874682 PMC220930

[bib10] Caplin ME, Pavel M, Ćwikła JB, et al. 2014 Lanreotide in metastatic enteropancreatic neuroendocrine tumors. N Engl J Med 371 224–233. (10.1056/nejmoa1316158) 25014687

[bib11] Capodanno Y, Buishand FO, Pang LY, et al. 2018 Notch pathway inhibition targets chemoresistant insulinoma cancer stem cells. Endocr Relat Cancer 25 131–144. (10.1530/erc-17-0415) 29175872

[bib12] Capodanno Y, Altieri B, Elders R, et al. 2022 Canine insulinoma as a model for human malignant insulinoma research: novel perspectives for translational clinical studies. Transl Oncol 15 101269. (10.1016/j.tranon.2021.101269) 34794032 PMC8605301

[bib13] Carney DN, Gazdar AF, Bepler G, et al. 1985 Establishment and identification of small cell lung cancer cell lines having classic and variant features. Cancer Res 45 2913–2923. 2985257

[bib14] Carter AM, Kumar N, Herring B, et al. 2021 Cdk5 drives formation of heterogeneous pancreatic neuroendocrine tumors. Oncogenesis 10 83. (10.1038/s41389-021-00372-5) 34862365 PMC8642406

[bib15] Chauhan A, Kohn E & Del Rivero J 2020 Neuroendocrine tumors-less well known, often misunderstood, and rapidly growing in incidence. JAMA Oncol 6 21–22. (10.1001/jamaoncol.2019.4568) 31697337 PMC9382718

[bib16] Choi S, Chen Z, Tang LH, et al. 2016 Bcl-xL promotes metastasis independent of its anti-apoptotic activity. Nat Commun 7 10384. (10.1038/ncomms10384) 26785948 PMC4735924

[bib17] Choi S, Wang D, Chen X, et al. 2019 Function and clinical relevance of RHAMM isoforms in pancreatic tumor progression. Mol Cancer 18 92. (10.1186/s12943-019-1018-y) 31072393 PMC6506944

[bib18] Clevers H 2016 Modeling development and disease with organoids. Cell 165 1586–1597. (10.1016/j.cell.2016.05.082) 27315476

[bib19] Crabtree JS, Scacheri PC, Ward JM, et al. 2001 A mouse model of multiple endocrine neoplasia, type 1, develops multiple endocrine tumors. Proc Natl Acad Sci U S A 98 1118–1123. (10.1073/pnas.98.3.1118) 11158604 PMC14718

[bib20] Crabtree JS, Scacheri PC, Ward JM, et al. 2003 Of mice and MEN1: insulinomas in a conditional mouse knockout. Mol Cell Biol 23 6075–6085. (10.1128/mcb.23.17.6075-6085.2003) 12917331 PMC180910

[bib21] D'Agosto S, Fiorini E, Pezzini F, et al. 2023 Long-term organoid culture of a small intestinal neuroendocrine tumor. Front Endocrinol 14 999792. (10.3389/fendo.2023.999792) PMC10112019 37082125

[bib22] Das S & Dasari A 2021 Epidemiology, incidence, and prevalence of neuroendocrine neoplasms: are there global differences? Curr Oncol Rep 23 43. (10.1007/s11912-021-01029-7) 33719003 PMC8118193

[bib23] Dayton TL, Alcala N, Moonen L, et al. 2023 Druggable growth dependencies and tumor evolution analysis in patient-derived organoids of neuroendocrine neoplasms from multiple body sites. Cancer Cell 41 2083–2099.e9. (10.1016/j.ccell.2023.11.007) 38086335

[bib24] Di Domenico A, Wiedmer T, Marinoni I, et al. 2017 Genetic and epigenetic drivers of neuroendocrine tumours (NET). Endocr Relat Cancer 24 R315–r334. (10.1530/erc-17-0012) 28710117

[bib25] Du YC, Lewis BC, Hanahan D, et al. 2007 Assessing tumor progression factors by somatic gene transfer into a mouse model: Bcl-xL promotes islet tumor cell invasion. PLoS Biol 5 e276. (10.1371/journal.pbio.0050276) 17941720 PMC2020504

[bib26] Du YC, Chou CK, Klimstra DS, et al. 2011 Receptor for hyaluronan-mediated motility isoform B promotes liver metastasis in a mouse model of multistep tumorigenesis and a tail vein assay for metastasis. Proc Natl Acad Sci U S A 108 16753–16758. (10.1073/pnas.1114022108) 21940500 PMC3189086

[bib27] Ear PH, Li G, Wu M, et al. 2019 Establishment and characterization of small bowel neuroendocrine tumor spheroids. J Vis Exp 152 60303. (10.3791/60303) PMC7654522 31657801

[bib28] Evers BM, Townsend CM Jr, Upp JR, et al. 1991 Establishment and characterization of a human carcinoid in nude mice and effect of various agents on tumor growth. Gastroenterology 101 303–311. (10.1016/0016-5085(91)90004-5) 1712329

[bib29] Fior R, Póvoa V, Mendes RV, et al. 2017 Single-cell functional and chemosensitive profiling of combinatorial colorectal therapy in zebrafish xenografts. Proc Natl Acad Sci U S A 114 E8234–E8243. (10.1073/pnas.1618389114) 28835536 PMC5625889

[bib30] Forsythe SD, Pu T, Andrews SG, et al. 2023 Models in pancreatic neuroendocrine neoplasms: current perspectives and future directions. Cancers 15 3756. (10.3390/cancers15153756) 37568572 PMC10416968

[bib31] Francis JM, Kiezun A, Ramos AH, et al. 2013 Somatic mutation of CDKN1B in small intestine neuroendocrine tumors. Nat Genet 45 1483–1486. (10.1038/ng.2821) 24185511 PMC4239432

[bib32] Frost M, Lines KE & Thakker RV 2018 Current and emerging therapies for PNETs in patients with or without MEN1. Nat Rev Endocrinol 14 216–227. (10.1038/nrendo.2018.3) 29449689 PMC6538535

[bib33] Fuentes ME, Lu X, Flores NM, et al. 2024 Combined deletion of MEN1, ATRX and PTEN triggers development of high-grade pancreatic neuroendocrine tumors in mice. Sci Rep 14 8510. (10.1038/s41598-024-58874-2) 38609433 PMC11014914

[bib34] Fujii M, Shimokawa M, Date S, et al. 2016 A colorectal tumor organoid library demonstrates progressive loss of niche factor requirements during tumorigenesis. Cell Stem Cell 18 827–838. (10.1016/j.stem.2016.04.003) 27212702

[bib35] Gape PMD, Schultz MK, Stasiuk GJ, et al. 2024 Towards effective targeted alpha therapy for neuroendocrine tumours: a review. Pharmaceuticals 17 334. (10.3390/ph17030334) 38543120 PMC10974115

[bib36] Gillette AA, Babiarz CP, VanDommelen AR, et al. 2021 Autofluorescence imaging of treatment response in neuroendocrine tumor organoids. Cancers 13 1873. (10.3390/cancers13081873) 33919802 PMC8070804

[bib37] Graham FL, Russell WC, Smiley J, et al. 1977 Characteristics of a human cell line transformed by DNA from human adenovirus type 5. J Gen Virol 36 59–72. (10.1099/0022-1317-36-1-59) 886304

[bib38] Greene LA & Tischler AS 1976 Establishment of a noradrenergic clonal line of rat adrenal pheochromocytoma cells which respond to nerve growth factor. Proc Natl Acad Sci U S A 73 2424–2428. (10.1073/pnas.73.7.2424) 1065897 PMC430592

[bib39] Hanahan D 1985 Heritable formation of pancreatic β-cell tumours in transgenic mice expressing recombinant insulin/simian virus 40 oncogenes. Nature 315 115–122. (10.1038/315115a0) 2986015

[bib40] Hanahan D, Christofori G, Naik P, et al. 1996 Transgenic mouse models of tumour angiogenesis: the angiogenic switch, its molecular controls, and prospects for preclinical therapeutic models. Eur J Cancer 32 2386–2393. (10.1016/s0959-8049(96)00401-7) 9059326

[bib41] Harding B, Lemos MC, Reed AA, et al. 2009 Multiple endocrine neoplasia type 1 knockout mice develop parathyroid, pancreatic, pituitary and adrenal tumours with hypercalcaemia, hypophosphataemia and hypercorticosteronaemia. Endocr Relat Cancer 16 1313–1327. (10.1677/erc-09-0082) 19620250 PMC4439740

[bib42] Herring B, Jang S, Whitt J, et al. 2021 *Ex vivo* modeling of human neuroendocrine tumors in tissue surrogates. Front Endocrinol 12 710009. (10.3389/fendo.2021.710009) PMC8734644 35002949

[bib43] Hofving T, Arvidsson Y, Almobarak B, et al. 2018 The neuroendocrine phenotype, genomic profile and therapeutic sensitivity of GEPNET cell lines. Endocr Relat Cancer 25 X1–X2. (10.1530/erc-17-0445e) 29540494 PMC8133373

[bib44] Jiao Y, Shi C, Edil BH, et al. 2011 DAXX/ATRX, MEN1, and mTOR pathway genes are frequently altered in pancreatic neuroendocrine tumors. Science 331 1199–1203. (10.1126/science.1200609) 21252315 PMC3144496

[bib45] Kaku M, Nishiyama T, Yagawa K, et al. 1980 Establishment of a carcinoembryonic antigen-producing cell line from human pancreatic carcinoma. Gan 71 596–601. 7227711

[bib46] Karna B, Pellegata NS & Mohr H 2024 Animal and cell culture models of PPGLs – achievements and limitations. Horm Metab Res 56 51–64. (10.1055/a-2204-4549) 38171372

[bib47] Kawasaki K, Toshimitsu K, Matano M, et al. 2020 An organoid biobank of neuroendocrine neoplasms enables genotype-phenotype mapping. Cell 183 1420–1435.e21. (10.1016/j.cell.2020.10.023) 33159857

[bib48] Kölby L, Bernhardt P, Ahlman H, et al. 2001 A transplantable human carcinoid as model for somatostatin receptor-mediated and amine transporter-mediated radionuclide uptake. Am J Pathol 158 745–755. (10.1016/s0002-9440(10)64017-5) 11159212 PMC1850312

[bib49] Kopper O, de Witte CJ, Lõhmussaar K, et al. 2019 An organoid platform for ovarian cancer captures intra- and interpatient heterogeneity. Nat Med 25 838–849. (10.1038/s41591-019-0422-6) 31011202

[bib50] Lee M, Marinoni I, Irmler M, et al. 2013 Transcriptome analysis of MENX-associated rat pituitary adenomas identifies novel molecular mechanisms involved in the pathogenesis of human pituitary gonadotroph adenomas. Acta Neuropathol 126 137–150. (10.1007/s00401-013-1132-7) 23756599 PMC3690182

[bib51] Lee D, Li M, Liu D, et al. 2024 Structural modifications toward improved lead-203/lead-212 peptide-based image-guided alpha-particle radiopharmaceutical therapies for neuroendocrine tumors. Eur J Nucl Med Mol Imaging 51 1147–1162. (10.1007/s00259-023-06494-9) 37955792 PMC10881741

[bib52] Lines KE, Stevenson M & Thakker RV 2016 Animal models of pituitary neoplasia. Mol Cell Endocrinol 421 68–81. (10.1016/j.mce.2015.08.024) 26320859 PMC4721536

[bib53] Lines KE, Vas Nunes RP, Frost M, et al. 2017 A MEN1 pancreatic neuroendocrine tumour mouse model under temporal control. Endocr Connect 6 232–242. (10.1530/ec-17-0040) 28420716 PMC5632719

[bib54] Loffler KA, Biondi CA, Gartside M, et al. 2007 Broad tumor spectrum in a mouse model of multiple endocrine neoplasia type 1. Int J Cancer 120 259–267. (10.1002/ijc.22288) 17044021

[bib55] Lou X, Ye Z, Xu X, et al. 2022 Establishment and characterization of the third non-functional human pancreatic neuroendocrine tumor cell line. Hum Cell 35 1248–1261. (10.1007/s13577-022-00696-3) 35394261

[bib56] Luley KB, Biedermann SB, Künstner A, et al. 2020 A comprehensive molecular characterization of the pancreatic neuroendocrine tumor cell lines BON-1 and QGP-1. Cancers 12 691. (10.3390/cancers12030691) 32183367 PMC7140066

[bib57] Maharjan CK, Ear PH, Tran CG, et al. 2021 Pancreatic neuroendocrine tumors: molecular mechanisms and therapeutic targets. Cancers 13 5117. (10.3390/cancers13205117) 34680266 PMC8533967

[bib58] Marques IJ, Weiss FU, Vlecken DH, et al. 2009 Metastatic behaviour of primary human tumours in a zebrafish xenotransplantation model. BMC Cancer 9 128. (10.1186/1471-2407-9-128) 19400945 PMC2697170

[bib59] Miyazaki J, Araki K, Yamato E, et al. 1990 Establishment of a pancreatic β cell line that retains glucose-inducible insulin secretion: special reference to expression of glucose transporter isoforms. Endocrinology 127 126–132. (10.1210/endo-127-1-126) 2163307

[bib60] Molatore S, Liyanarachchi S, Irmler M, et al. 2010 Pheochromocytoma in rats with multiple endocrine neoplasia (MENX) shares gene expression patterns with human pheochromocytoma. Proc Natl Acad Sci U S A 107 18493–18498. (10.1073/pnas.1003956107) 20937862 PMC2972990

[bib61] Monazzam A, Li S-C, Wargelius H, et al. 2020 Generation and characterization of CRISPR/Cas9-mediated MEN1 knockout BON1 cells: a human pancreatic neuroendocrine cell line. Sci Rep 10 14572. (10.1038/s41598-020-71516-7) 32884006 PMC7471701

[bib62] O'Dorisio TM, Harris AG & O'Dorisio MS 2020 Evolution of neuroendocrine tumor therapy. Surg Oncol Clin N Am 29 145–163. (10.1016/j.soc.2019.11.002) 32151353 PMC7702714

[bib63] Pellegata NS, Quintanilla-Martinez L, Siggelkow H, et al. 2006 Germ-line mutations in p27 *Kip1* cause a multiple endocrine neoplasia syndrome in rats and humans. Proc Natl Acad Sci U S A 103 15558–15563. (10.1073/pnas.0603877103) 17030811 PMC1622862

[bib64] Piotrowska K, S Pellegata N, Rosemann M, et al. 2004 Mapping of a novel MEN-like syndrome locus to rat chromosome 4. Mamm Genome 15 135–141. (10.1007/s00335-003-3027-8) 15058384

[bib65] Puck TT & Marcus PI 1955 A rapid method for viable cell titration and clone production with hela cells in tissue culture: the use of X-irradiated cells to supply conditioning factors. Proc Natl Acad Sci U S A 41 432–437. (10.1073/pnas.41.7.432) 16589695 PMC528114

[bib66] Raymond E, Dahan L, Raoul JL, et al. 2011 Sunitinib malate for the treatment of pancreatic neuroendocrine tumors. N Engl J Med 364 501–513. (10.1056/nejmoa1003825) 21306237

[bib67] Riihimäki M, Hemminki A, Sundquist K, et al. 2016 The epidemiology of metastases in neuroendocrine tumors. Int J Cancer 139 2679–2686. (10.1002/ijc.30400) 27553864

[bib68] Rindi G, Klimstra DS, Abedi-Ardekani B, et al. 2018 A common classification framework for neuroendocrine neoplasms: an International Agency for Research on Cancer (IARC) and World Health Organization (WHO) expert consensus proposal. Mod Pathol 31 1770–1786. (10.1038/s41379-018-0110-y) 30140036 PMC6265262

[bib69] Rinke A, Müller HH, Schade-Brittinger C, et al. 2009 Placebo-controlled, double-blind, prospective, randomized study on the effect of octreotide LAR in the control of tumor growth in patients with metastatic neuroendocrine midgut tumors: a report from the PROMID study group. J Clin Oncol 27 4656–4663. (10.1200/jco.2009.22.8510) 19704057

[bib70] Roeyen G, Chapelle T, Borbath I, et al. 2009 The role of surgery and transplantation in neuroendocrine tumours. Acta Gastroenterol Belg 72 39–43. 19402370

[bib71] Scarpa A, Chang DK, Nones K, et al. 2017 Whole-genome landscape of pancreatic neuroendocrine tumours. Nature 543 65–71. (10.1038/nature21063) 28199314

[bib72] Sedlack AJH, Saleh-Anaraki K, Kumar S, et al. 2022 Preclinical models of neuroendocrine neoplasia. Cancers 14 5646. (10.3390/cancers14225646) 36428741 PMC9688518

[bib73] Singh S, Halperin D, Myrehaug S, et al. 2024 [(177)Lu]Lu-DOTA-TATE plus long-acting octreotide versus high-dose long-acting octreotide for the treatment of newly diagnosed, advanced grade 2-3, well-differentiated, gastroenteropancreatic neuroendocrine tumours (NETTER-2): an open-label, randomised, phase 3 study. Lancet 403 2807–2817. (10.1016/s0140-6736(24)00701-3) 38851203

[bib74] Skelin M, Rupnik M & Cencic A 2010 Pancreatic beta cell lines and their applications in diabetes mellitus research. Altex 27 105–113. (10.14573/altex.2010.2.105) 20686743

[bib75] Strosberg J, El-Haddad G, Wolin E, et al. 2017 Phase 3 trial of (177)Lu-dotatate for midgut neuroendocrine tumors. N Engl J Med 376 125–135. (10.1056/nejmoa1607427) 28076709 PMC5895095

[bib76] Sun C, Estrella JS, Whitley EM, et al. 2022 Context matters - Daxx and Atrx are not robust tumor suppressors in the murine endocrine pancreas. Dis Model Mech 15 dmm049552. (10.1242/dmm.049552) 35976056 PMC9438929

[bib77] Telerman A, Yossef Y, Chmelnik A, et al. 2023 THU487 xenograft of VHL-deficient pancreatic neuroendocrine neoplasm cells – a novel low-grade PNEN in vivo model. J Endocr Soc 7 bvad114.2115. (10.1210/jendso/bvad114.2115)

[bib78] Tillotson LG, Lodestro C, Höcker M, et al. 2001 Isolation, maintenance, and characterization of human pancreatic islet tumor cells expressing vasoactive intestinal peptide. Pancreas 22 91–98. (10.1097/00006676-200101000-00016) 11138979

[bib79] Tran CG, Borbon LC, Mudd JL, et al. 2022 Establishment of novel neuroendocrine carcinoma patient-derived xenograft models for receptor peptide-targeted therapy. Cancers 14 1910. (10.3390/cancers14081910) 35454817 PMC9033026

[bib80] van Riet J, van de Werken HJG, Cuppen E, et al. 2021 The genomic landscape of 85 advanced neuroendocrine neoplasms reveals subtype-heterogeneity and potential therapeutic targets. Nat Commun 12 4612. (10.1038/s41467-021-24812-3) 34326338 PMC8322054

[bib81] Vandamme T, Peeters M, Dogan F, et al. 2015 Whole-exome characterization of pancreatic neuroendocrine tumor cell lines BON-1 and QGP-1. J Mol Endocrinol 54 137–147. (10.1530/jme-14-0304) 25612765

[bib82] Viol F, Sipos B, Fahl M, et al. 2022 Novel preclinical gastroenteropancreatic neuroendocrine neoplasia models demonstrate the feasibility of mutation-based targeted therapy. Cell Oncol 45 1401–1419. (10.1007/s13402-022-00727-z) PMC9747820 36269546

[bib83] Vitale G, Gaudenzi G, Dicitore A, et al. 2014 Zebrafish as an innovative model for neuroendocrine tumors. Endocr Relat Cancer 21 R67–R83. (10.1530/erc-13-0388) 24292602

[bib84] Wasylishen AR, Sun C, Moyer SM, et al. 2020 Daxx maintains endogenous retroviral silencing and restricts cellular plasticity in vivo. Sci Adv 6 eaba8415. (10.1126/sciadv.aba8415) 32821827 PMC7406367

[bib85] White BE, Rous B, Chandrakumaran K, et al. 2022 Incidence and survival of neuroendocrine neoplasia in England 1995–2018: a retrospective, population-based study. Lancet Reg Health Eur 23 100510. (10.1016/j.lanepe.2022.100510) 36176500 PMC9513765

[bib87] Yao JC, Shah MH, Ito T, et al. 2011 Everolimus for advanced pancreatic neuroendocrine tumors. N Engl J Med 364 514–523. (10.1056/nejmoa1009290) 21306238 PMC4208619

[bib86] Yao JC, Fazio N, Singh S, et al. 2016 Everolimus for the treatment of advanced, non-functional neuroendocrine tumours of the lung or gastrointestinal tract (RADIANT-4): a randomised, placebo-controlled, phase 3 study. Lancet 387 968–977. (10.1016/s0140-6736(15)00817-x) 26703889 PMC6063317

[bib88] Zandee WT & de Herder WW 2018 The evolution of neuroendocrine tumor treatment reflected by ENETS guidelines. Neuroendocrinology 106 357–365.(10.1159/000486096) 29320780 PMC6067804

[bib89] Zhang G, Chi Y & Du YN 2017 Identification and characterization of metastatic factors by gene transfer into the novel RIP-tag; RIP-tva murine model. J Vis Exp 128 55890. (10.3791/55890) PMC5644377 29155705

[bib90] Zhang T, Choi S, Zhang T, et al. 2020 miR-431 promotes metastasis of pancreatic neuroendocrine tumors by targeting DAB2 interacting protein, a Ras GTPase activating protein tumor suppressor. Am J Pathol 190 689–701. (10.1016/j.ajpath.2019.11.007) 31953039 PMC7074368

[bib91] Zhu Z, Cui W, Zhu D, et al. 2020 Common tools for pituitary adenomas research: cell lines and primary cells. Pituitary 23 182–188. (10.1007/s11102-019-01003-4) 31728905

